# Acute Toxicity of Dinotefuran to *Picromerus lewisi* Scott (Hemiptera: Pentatomidae) and Its Impact on Offspring Growth and Predation Ability in Integrated Pest Management

**DOI:** 10.3390/insects16040404

**Published:** 2025-04-11

**Authors:** Yutong Ji, Mengqing Wang, Chuanzhen Xue, Jianjun Mao, Yuyan Li, Lisheng Zhang

**Affiliations:** State Key Laboratory for Biology of Plant Diseases and Insect Pests, Key Laboratory of Natural Enemy Insects, Ministry of Agriculture and Rural Affairs, Institute of Plant Protection, Chinese Academy of Agricultural Sciences, Beijing 100193, China; jiyutong59@163.com (Y.J.); xchuanzhen@163.com (C.X.); maojianjun0615@126.com (J.M.); liyuyan@caas.cn (Y.L.); zhanglisheng@caas.cn (L.Z.)

**Keywords:** *Picromerus lewisi*, dinotefuran, LC_50_, offspring, biological control, integrated pest management

## Abstract

In integrated pest management (IPM), achieving a balance between biological and chemical control strategies is essential for effective pest control. Therefore, it is crucial to assess the impact of chemical pesticides on natural enemies. Neonicotinoid insecticides, particularly dinotefuran, are among the most widely used insecticides globally. This study investigated the acute toxicity of dinotefuran to adult predatory natural enemy *Picromerus lewisi* and evaluated its effects on offspring growth and predation. The results showed that the 72 h median lethal concentrations (LC_50_) of dinotefuran for female and male adults (F_0_) were 0.624 mg/L and 0.592 mg/L, respectively. Exposure to LC_50_ concentrations of dinotefuran significantly reduced parental fecundity, longevity, and offspring growth and predatory ability. The most pronounced effects were observed when both female and male adults were exposed. Specifically, the pre-oviposition period was 2.64 times longer than in the control group, oviposition frequency dropped to 18.6%, total fecundity decreased to 13.4%, and the offspring emergence rate was reduced to 50% of the control group’s level. Furthermore, predation efficacy of F_1_ generation (5th instar nymphs and adults) on *Spodoptera exigua* and *Spodoptera litura* larvae reduced by 52.76% to 66.81%. Exposure of only female adults also led to significant but less severe effects on offspring, while male-only exposure showed the least impact. These findings highlighted the toxicological risks of dinotefuran to *P. lewisi*, impairing both reproductive and predatory functions, which could undermine biological control and affect the success of IPM strategies.

## Simple Summary

Biological control has become a key component of integrated pest management (IPM) and is essential for sustainable agricultural development. Chemical control remains another important pest management strategy in agriculture. Neonicotinoid insecticides, particularly dinotefuran, are among the most widely used insecticides worldwide. Therefore, achieving a balance between biological and chemical control strategies is critical for effective pest management. This study investigates the acute toxicity of dinotefuran to both female and male adult *Picromerus lewisi*, a predatory natural enemy, and evaluates its effects on offspring growth and predation. Our results show that exposure of both female and male adults to the half-lethal concentration (LC_50_) of dinotefuran has the most significant impact on offspring, with female exposure alone also leading to substantial effects, while male exposure has the least impact. These findings highlight the toxicological risks of dinotefuran to *P. lewisi*, providing deeper insights into its effects on non-target organisms.

## 1. Introduction

Pesticides are commonly used for pest control due to their rapid action, high efficiency, and cost-effectiveness. However, their improper use, overuse, or misuse can lead to pesticide residues that pose significant risks to human health and contribute to environmental contamination [1]. As societal awareness increases and public demand for safe, sustainable food grows, biological control methods—recognized for their safety and environmental compatibility—are gaining popularity as an alternative to chemical pesticides. These methods offer a potential solution to mitigate the negative impacts of chemical control. Consequently, biological control has become a key component of integrated pest management (IPM) and is increasingly considered essential for sustainable agricultural practices. Among the various biological control strategies, the use of natural enemies of pests has received considerable attention [2].

Understanding the impact of chemical pesticides on natural enemies is crucial for promoting their rational use, minimizing harm to beneficial organisms, and integrating chemical and biological control methods. This knowledge provides a scientific basis for balancing these control strategies [3,4]. The effects of chemical pesticides on natural enemies can be categorized into two types: direct effects and indirect effects, which include food chain toxicity (secondary poisoning) and sub-lethal impacts [5,6]. For instance, Bredeson et al. (2015) [7] demonstrated that second-instar ladybugs (*Coleomegilla maculata*) exhibited slower walking behavior when feeding on aphids (*Rhopalosiphum padi*) on wheat plants treated with thiamethoxam. Cheng et al. (2018) [8] reported that the LR_50_ of acetamiprid for adult *Amblyseius cucumeris* was 76.36 g a.i./ha. In addition, exposure to sublethal concentrations of acetamiprid significantly prolonged the larval stage of *Amblyseius cucumeris*. These studies underscore the differential toxicological effects of various pesticides on natural enemies.

Pesticides also affect the development, mobility, and predatory behavior of natural enemies [9]. For instance, Lima et al. (2020) [10] observed that the application of deltamethrin to third-instar nymphs of *Podisus nigrispinus* significantly increased both their crawling speed and distance. Passos et al. (2018) [11] demonstrated that exposure of *Macrolophus basicornis* to teflubenzuron and methoxyfenozide significantly reduced the hind tibia length of females during the nymphal stage. Additionally, Jiang et al. (2019) [12] reported that at the LC_10_ concentration of thiamethoxam, both adult emergence and reproductive capacity of *Coccinella septempunctata* were significantly decreased.

Additionally, the effects of pesticides on the predatory behavior and functional capacity of natural enemies have been extensively studied. Mostafiz et al. (2020) [13] exposed adult *Nesidiocoris tenuis* to varying concentrations of methyl benzoate and assessed their predation rate on *Bemisia tabaci* eggs. The results revealed that as the concentration of methyl benzoate increased, the predation rate of *N. tenuis* decreased progressively.

Dinotefuran, a third-generation neonicotinoid insecticide, is one of the most widely used insecticides globally [14]. Due to its strong systemic activity, long-lasting effects, and broad spectrum of action, dinotefuran is extensively applied [15]. It is particularly effective against lepidopteran pests and piercing–sucking insects. However, studies have raised concerns about its safety for biological control, human health, and the environment, particularly due to its high toxicity to non-target organisms such as bees and earthworms [16,17]. *Picromerus lewisi*, a valuable natural enemy of lepidopteran pests and piercing–sucking insects, is an important species in integrated pest management. This study aims to assess the acute toxicity of dinotefuran to adult male and female *P*. *lewisi*, and its effects on the growth and development of their offspring, to better understand its impact on non-target species.

## 2. Materials and Methods

### 2.1. Insecticides

The dinotefuran (98%) used in this experiment was purchased from Chengdu West Asia Chemical Co., Ltd., (Chengdu West Asia Chemical Co., Ltd., Chengdu, China). The solvent used by dissolving dinotefuran was HPLC-grade acetone (Shanghai McLean Biochemical Technology Co., Ltd., Shanghai, China).

### 2.2. Test Insects

Adult males and females of *Picromerus lewisi* were obtained from the Natural Enemy Insect Group at the Institute of Plant Protection, Chinese Academy of Agricultural Sciences, Haidian District, Beijing, China. The insects were reared on last-instar larvae of *Galleria mellonella* (Lepidoptera: Pyralidae), which were purchased from Xiaohouzi Information Technology Co., Ltd. (Xiaohouzi Information Technology Co., Ltd., Chongqing, China). The adults used in the experiments were allowed to molt for 48–72 h prior to testing. The third instar larvae of *Spodoptera exigua* and *Spodoptera litura* were purchased from Haokang Biotechnology Co., Ltd. (Haokang Biotechnology Co., Ltd., Jilin, China). All experiments were conducted under laboratory artificial constant temperature and humidity incubator conditions, with a temperature of 26 ± 1 °C, 70 ± 5% relative humidity, and a 16:8 light:dark photoperiod.

### 2.3. Bioassays

#### 2.3.1. Concentration Mortality Response in 72 h Toxicity Contact Test

The residual film method was used in this experiment. Based on the results of a preliminary test, a specified amount of dinotefuran TC was dissolved in acetone and serially diluted to create concentrations of 10, 5, 2.5, 1.25, 0.625, and 0.3125 mg/L to determine the half-lethal concentrations. These concentrations were chosen based on a 72 h acute toxicity experiment. For each concentration, 0.5 mL of the insecticide solution was added to a 12 cm high, 1.5 cm diameter glass culture tube, which was then sealed with gauze. The solution was evenly distributed in the tube, and a blank control using only acetone was included. The tubes were immediately spun to evaporate the solvent until dry.

In the preparatory test, seven treatments were set up, each with three replicates. One adult female or male *P. lewisi* was introduced into each replicate and provided with last-instar larvae of *G. mellonella* as a food source. Thirty test insects (either female or male adults) were inoculated into each concentration, with the number of *G*. *mellonella* larvae equal to the number of test insects. The secretions of *P. lewisi* and any carcasses of *G. mellonella* were removed daily, and fresh larvae were added to ensure an adequate food supply. Mortality of the adults was recorded after 72 h. To assess mortality, the *P. lewisi* individuals were gently touched with a soft brush. Individuals that did not respond (i.e., were immobile) were considered dead. The mortality rate of the control group was less than 10%, and the results were deemed valid [12].

#### 2.3.2. Half-Lethal Toxicity Bioassays

To assess the half-lethal effects of dinotefuran on parental adult females and males (F_0_) of *P. lewisi*, the LC_50_ values for females and males were determined through a concentration–mortality response.

Additionally, to evaluate the impact of the half-lethal concentrations of dinotefuran on the fecundity of the parental (F_0_) generation, as well as the development and predation ability of the progeny (F_1_), four treatments were established: Both treated (B.t.), Female treated (F.t.), Male treated (M.t.), and a control group (CK), each with ten replicates. In each treatment, one adult female and one adult male were paired and reared on last-instar larvae of *G. mellonella*. The fecundity of the females (including pre-oviposition duration, oviposition frequency, total fecundity, and hatching proportion of eggs), as well as the longevity of the parental (F_0_) individuals, were monitored and recorded every 12 h until all F_0_ individuals had died.

To further investigate the effects of dinotefuran on the progeny (F_1_), eggs newly laid by the parental individuals (F_0_) were collected from each treatment and placed in separate 10 cm diameter Petri dishes. The eggs were sprayed with water and checked daily until hatching was complete. Thirty newly hatched nymphs from each treatment were collected and individually placed into 250 mL plastic cups with water. The second-instar nymphs were reared on last-instar *G. mellonella* larvae until they reached adulthood. During the experiment, the hatching duration of the eggs, developmental duration, survival rate, and acquisition proportion of progeny (F_1_) under different treatments were recorded every 12 h. The predation ability of the fifth-instar nymphs, adult females, and males of the progeny (F_1_) in each treatment was also assessed. The experiment concluded when all progeny (F_1_) had reached adulthood.

### 2.4. Functional Response of the P. lewisi F_1_ Generation to S. exigua and S. litura

To investigate the functional response of *P. lewisi* offspring to *S. exigua* and *S. litura* under half-lethal exposure conditions, five prey densities (5, 10, 15, 20, and 40 individuals) of each species were offered to fifth-instar nymphs, adult females, and adult males of *P. lewisi* (F_1_) after 24 h of starvation. All experiments were conducted in incubators set at 26 ± 1 °C, 70 ± 5% relative humidity (RH), and a 16:8 (light:dark) photoperiod.

For each trial, the artificial diet and third-instar larvae of *S. exigua* or *S. litura* were placed on corn leaves in transparent, breathable boxes measuring 21 cm in length, 14 cm in width, and 9 cm in height. Each fifth-instar nymph, adult female, and adult male *P. lewisi* was tested in triplicate, with 15 replicates for each prey density. The number of preys captured was recorded after 24 h.

### 2.5. Data Analysis

All data were analyzed using Excel 2019. Log-probit regression analysis was performed using SPSS software (Version 21.0, SPSS Inc., Chicago, IL, USA) to determine the LC_50_ (the concentration causing 50% mortality of the test species). The 72 h mortality of adult females and males of *P. lewisi* (F_0_) during the LC_50_ determination was analyzed using chi-square tests. The half-lethal effects of dinotefuran on the parental generation of *P. lewisi* were analyzed using one-way analysis of variance (ANOVA), with multiple comparisons between treatment groups conducted using Tukey’s honest significant difference (HSD) test (*p* < 0.05). Graphs were generated using Origin 2018.

Holling classified functional responses into three types: Type I, Type II, and Type III [18]. Type II and Type III functional responses are dominant in predator–prey dynamics studies, characterized by curvilinear (Type II) and sigmoidal (Type III) scaling of per capita predation rates relative to prey density [19]. Additionally, based on our laboratory previous studies on the predatory behavior of *P. lewisi* toward *S. frugiperda*, we have adopted the Holling Type II functional response to analyze the predation data for this study. The data were fitted to Holling’s DISC equation [18]: *N_a_* = *aNT_r_*/(1 + *aNT_h_*). Where *N_a_* is the number of preys consumed, *a* is the instantaneous attack rate, *N* is the initial prey density, *T_r_* is the duration of the test (24 h, i.e., *T_r_* = 1 day), and *T_h_* is the handling time (the time each predator takes to capture, attack, and consume a single prey). The search efficiency “*S*” was calculated using the equation: *S* = *aT_r_*/(1 + *aT_h_N*) [20]. Prey density is a determinant of search efficiency, and the searching efficiency of predators is negatively correlated with prey density. One-way analysis of variance (ANOVA) was used to analyze the effects of dinotefuran on the predation function of *P. lewisi* progeny, with Turkey’s HSD test applied for multiple comparisons between treatment groups (*p* < 0.05). Graphs were generated using Origin 2018 and GraphPad Prism 8.0.2.

## 3. Results

### 3.1. Effects of 72 h Toxicity Contact Test

Log-probit regression analysis revealed that the LC_25_ of dinotefuran for adult female *P. lewisi* was 0.184 mg L^−1^, with a 95% confidence interval (CI) of 0.081–0.295 mg L^−1^. The LC_50_ of dinotefuran for adult female *P. lewisi* was 0.624 mg L^−1^, with a 95% confidence interval (CI) of 0.421–0.833 mg L^−1^. The LC_90_ of dinotefuran for adult female *P. lewisi* was 4.766 mg L^−1^, with a 95% confidence interval (CI) of 3.410–7.745 mg L^−1^. The regression equation obtained from the generalized linear model fitting was *y* = 1.564*x* + 0.356 (*x*^2^ = 0.755, *df* = 3, *p* = 0.860). This indicates a good fit of the regression model. The standard errors (SE) of the slope and intercept were 0.192 and 0.078, respectively.

The LC_25_ of dinotefuran for adult male *P. lewisi* was 0.219 mg L^−1^, with a 95% confidence interval (CI) of 0.121–0.319 mg L^−1^. The LC_50_ of dinotefuran for adult male *P. lewisi* was 0.592 mg L^−1^, with a 95% confidence interval (CI) of 0.430–0.756 mg L^−1^. The LC_90_ of dinotefuran for adult male *P. lewisi* was 3.497 mg L^−1^, with a 95% confidence interval (CI) of 2.551–5.563 mg L^−1^. The regression equation derived from the linear model fitting was *y* = 1.269*x* + 0.259 (*x*^2^ = 0.957, *df* = 3, *p* = 0.812). This indicates a good fit of the regression model. The standard errors (SE) of the slope and intercept were 0.207 and 0.081, respectively.

### 3.2. Effects of Dinotefuran on Parental (F_0_) Fecundity and Longevity of P. lewisi

The LC_50_ of dinotefuran significantly affected the fecundity of the parental generation. The pre-oviposition duration, oviposition frequency, and total fecundity in each treatment were significantly different from those in CK, with notable differences among the three treatments as well.

**Figure 1:**
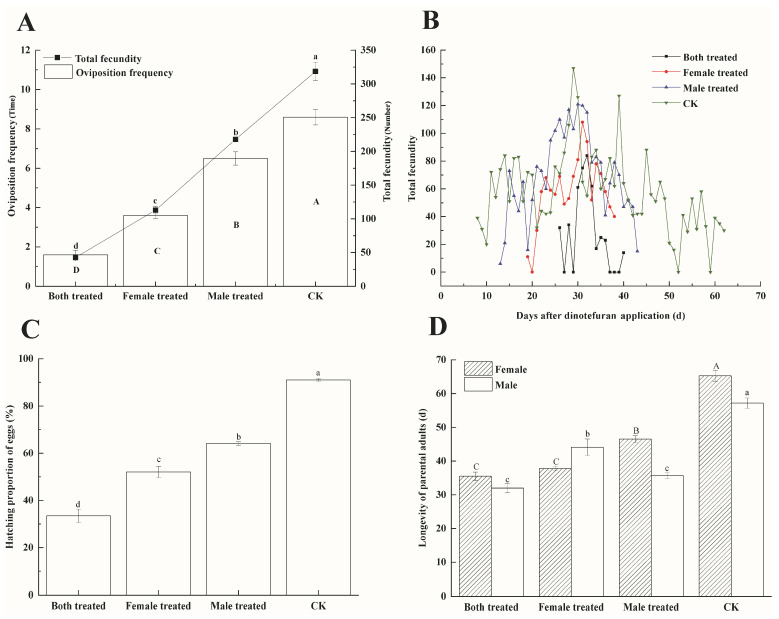
Oviposition frequency, fecundity, hatching proportion of eggs and longevity of *P. lewisi* parental (F_0_) under dinotefuran. Different letters indicated statistically differences (*p* < 0.05) according to Turkey’s HSD Test (Compare uppercase letters with each other and compare lowercase letters with each other). (**A**): Oviposition frequency and fecundity of *P. lewisi* parental (F_0_) under dinotefuran. (**B**): Oviposition of *P. lewisi* parental (F_0_) under dinotefuran. (**C**): Hatching proportion of *P. lewisi* parental (F_0_) eggs under dinotefuran. (**D**): Longevity of *P. lewisi* parental (F_0_) under dinotefuran.

The pre-oviposition duration in the B.t. group was 30.40 ± 0.65 days, the longest of all treatments, and 2.64 times longer than the CK (*F* = 575.31, *df* = 3, *p* < 0.01). The oviposition frequency was 1.60 ± 0.22 times, significantly lower than the other two treatments and CK, and only 18.60% of that in the CK (*F* = 108.67, *df* = 3, *p* < 0.01). Fecundity in the B.t. group was 42.7 ± 5.02 eggs, significantly lower than in the other treatments and CK, representing 13.41% of the CK value (*F* = 3.02, *df* = 3, *p* < 0.05) (Table 1, Figure 1A).

Figure 1B illustrated the oviposition patterns of all tested *P. lewisi* parental individuals. The shortest oviposition duration in the B.t. group was 15 days, with a maximum daily fecundity of 84 eggs. In the F.t. group, the oviposition duration was 20 days, and the maximum daily fecundity was 108 eggs. The M.t. group exhibited a 31-day oviposition duration, with a maximum daily fecundity of 121 eggs. The CK group had the longest oviposition duration of 55 days, with a maximum daily fecundity of 147 eggs.

The LC_50_ of dinotefuran also significantly impacted the hatching proportion of *P. lewisi* eggs. The hatching proportions in all treatments were significantly different from the CK, with significant differences observed among the three treatments. The hatching proportion in the B.t. group was 33.48 ± 2.85%, significantly lower than in the other treatments and the CK. In contrast, the hatching proportions in the F.t. group, M.t. group, and CK were 52.02 ± 2.37%, 64.13 ± 0.73%, and 90.97 ± 0.48%, respectively (*F* = 73.56, *df* = 3, *p* < 0.01) (Figure 1C).

Figure 1D showed the effects of dinotefuran on the longevity of the *P. lewisi* parental generation (Female: *F* = 132.86, *df* = 3, *p* < 0.01. Male: *F* = 47.23, *df* = 3, *p* < 0.01). Adult females in the B.t. group lived the shortest (35.50 ± 1.26 days), which was 0.76 times that of the M.t. group and 0.54 times that of the CK group. However, no significant difference was observed between the B.t. and F.t. groups (37.80 ± 0.57 days). The longevity of adult females in the M.t. group was significantly shorter than those in the CK group. Similarly, adult males in the B.t. group lived the shortest amount of time (32.00 ± 1.30 days), 0.73 times the longevity of the F.t. group and 0.56 times that of the CK group, with no significant difference between the B.t. and M.t. groups (35.70 ± 0.84 days). The longevity of adult males in the F.t. group was significantly shorter than in the CK group. In both the B.t. and CK groups, adult females lived longer than adult males when treated identically, while adult males in the F.t. group lived longer than females without treatment.

### 3.3. Effects of Dinotefuran on Progeny (F_1_) Developmental Duration and Survival of P. lewisi

**Figure 2:**
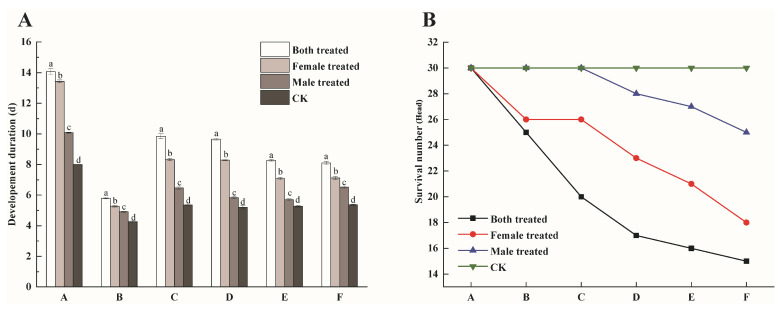
Hatching duration of *P. lewisi* eggs, developmental duration (**A**) and survival number of *P. lewisi* progeny (F_1_) under dinotefuran (**B**) (A: Eggs; B: First instar nymphs; C: Second instar nymphs; D: Third instar nymphs; E: Fourth instar nymphs; F: Fifth instar nymphs). Different letters indicated statistically differences (*p* < 0.05) according to Turkey’s HSD Test.

The results showed that dinotefuran significantly affected the hatching duration of eggs (*F*_3, 116_ = 743.70, *p* < 0.001). The B.t. group had the longest hatching duration (14.07 ± 0.19 days), which was significantly longer than the other treatments and CK, representing 1.76 times that of CK. Dinotefuran also had significant effects on the developmental duration of *P. lewisi* nymphs across all instars: the 1st instar (*F*_3, 116_ = 218.80, *p* < 0.001), the 2nd instar (*F*_3, 107_ = 413.60, *p* < 0.001), the 3rd instar (*F*_3, 102_ = 748.70, *p* < 0.001), the 4th instar (*F*_3, 94_ = 428.40, *p* < 0.001) and the 5th instar (*F*_3, 90_ = 242.30, *p* < 0.001). In the B.t. group, the developmental durations for each instar were the longest: 5.80 ± 0.05 days for the 1st instar, 9.84 ± 0.16 days for the 2nd instar, 9.65 ± 0.07 days for the 3rd instar, 8.27 ± 0.06 days for the 4th instar and 8.09 ± 0.10 days for the 5th instar. respectively. These durations were significantly longer than those in the other treatments and CK (Figure 2A).

As shown in Figure 2B, the survival rate of the nymphs in the B.t. group declined over time, with mortality occurring in all instars. The adult emergence proportion in the B.t. group (50%) was the lowest among all treatments, half that of CK, and the female-to-male sex ratio was 1:2. In the F.t. group, the 2nd instar nymphs survived, but all other instars died. The adult emergence proportion in the F.t. group was 60%, with a female-to-male sex ratio of 1:1.57. In the M.t. group, the 1st and 2nd instar nymphs survived, but the other instars died. The adult emergence proportion in the M.t. group was 83%, and the female-to-male sex ratio was 1:1.78. The adult emergence proportions across the groups were as follows: B.t. group < F.t. group < M.t. group < CK (Table 2).

### 3.4. Effects of Dinotefuran on Progeny (F_1_) Predation Functional Response of P. lewisi

**Figure 3:**
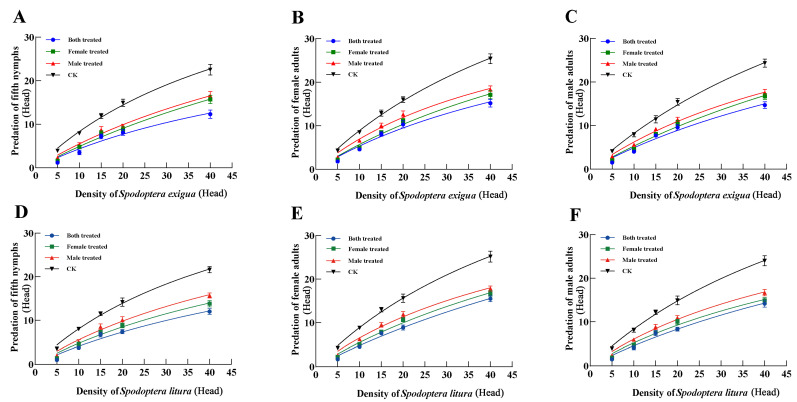
Functional responses of *P. lewisi* progeny (F_1_) to 3rd instar larvae of *S. exigua* (**A**–**C**) and *S. litura* under dinotefuran (**D**–**F**).

The predation functional response of the 5th instar nymphs, adult females, and adult males in the three treatments and CK to the 3rd instar larvae of *S. exigua* followed the Holling Type II model, with predation increasing as prey density rose. The instantaneous attack rate of the 5th instar nymphs and adult females and males in CK was the highest, recorded at 0.985, 1.024, and 0.925, respectively. In contrast, the B.t. group had the lowest attack rates, with values of 0.499, 0.613, and 0.545, respectively. The control efficiency (the proportion of prey consumed) of the 5th instar nymphs and adult females and males in CK was also the highest, with values of 51.842, 68.267, and 66.071, respectively. The B.t. group showed the weakest control efficiency, with values of 17.207, 25.542, and 25.952, respectively. For all treatments, the predation, instantaneous attack rate, and control efficiency ranked as follows: B.t. group < F.t. group < M.t. group < CK (Figure 3A–C, Table 3).

The predation functional response of the 5th instar nymphs and adult females and males of the three treatments and CK to the 3rd instar larvae of *S. litura* also conformed to the Holling Type II model, with predation increasing with higher prey density. The instantaneous attack rate of the 5th instar nymphs and adult females and males in CK was again the highest, at 0.958, 1.030 and 0.956, respectively, while the B.t. group exhibited the lowest attack rates of 0.459, 0.519, and 0.498, respectively. Similarly, the control efficiency in CK was the highest, at 47.900, 68.667, and 63.733, respectively, whereas the B.t. group showed the weakest control efficiency, with values of 16.393, 32.438, and 24.900, respectively. As with the *S. exigua* data, the predation, instantaneous attack rate, and control efficiency of each group were ranked: B.t. group < F.t. group < M.t. group < CK (Figure 3D–F, Table 4).

### 3.5. Effects of Dinotefuran on Progeny (F_1_) Searching Efficiency of P. lewisi

**Figure 4:**
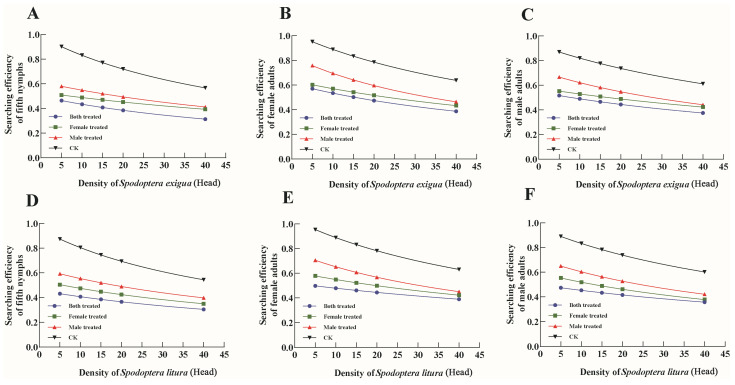
Searching efficiency of *P. lewisi* progeny (F_1_) to 3rd instar larvae of *S. exigua* (**A**–**C**) and *S. litura* (**D**–**F**) under dinotefuran.

The searching efficiency of the 5th instar nymphs, adult females, and adult males towards 3rd instar larvae of *S. exigua* and *S. litura* decreased with increasing prey density in all treatments and CK. When comparing the same instar of *P. lewisi*, the searching efficiency of each group followed this order: B.t. group < F.t. group < M.t. group < CK (Figure 4).

## 4. Discussion

The results of this study demonstrated that the LC_50_ of dinotefuran significantly impaired the reproductive capacity (e.g., pre-oviposition duration, oviposition frequency) and longevity of *P. lewisi* (F_0_). These findings align with previous research, which also reported that dinotefuran adversely affected the development and reproduction of natural enemies, such as *Trichogramma ostriniae* [21]. Furthermore, studies have shown that acetamiprid and imidacloprid reduce the survival rate of female adult *Orius sauteri* [22,23]. Furthermore, dinotefuran caused transgenerational effects, negatively affecting the development and predatory capacity of the F_1_ generation. Similarly, exposure to other neonicotinoids, such as thiamethoxam and imidacloprid, significantly reduced the emergence rate, reproductive capacity, and longevity of adult *Coccinella septempunctata*, while also impairing the predation activity of the F_1_ generation [12,24].

Among the three treatment groups in this study, the B.t. group exhibited the most significant effects on both the reproductive capacity and longevity of *P. lewisi* (F_0_), as well as on the development and predatory capacity of the F_1_ generation. Observations revealed that the interval from pesticide application to the initiation of autonomous feeding and mating was longest for the B.t. group, followed by the F.t. group, while the M.t. group showed the least impact. In the M.t. group, female adults were untreated, but their longevity was significantly shorter than that of the control group. This suggested that the observed effects may be attributed to mating or feeding on the same prey as male adults, which had been exposed to dinotefuran. Similarly, untreated male adults in the F.t. group exhibited similar effects, possibly due to the transfer of toxins between male and female insects through mating [25,26].

Our findings on oviposition frequency in the F.t. and M.t. groups align with those reported by Wang et al. (2010) [27]. When treated males mated with untreated females, the females exhibited a significantly higher oviposition frequency compared to when treated females mated with untreated males. This difference may be attributed to pesticide stress, which could interfere with ovarian development [28]. In the B.t. group, multiple instances of hollow or shriveled eggs were observed. Previous studies have indicated that pesticide stress can induce apoptosis in male insect testicular tissue, which in turn affects ovarian development in females. This disruption can lead to significant reductions in reproductive capacity [28,29].

Exposure to the LC_50_ concentration of dinotefuran impaired the reproductive capacity of both male and female adults, leading to the production of non-viable eggs. This resulted in reduced egg hatchability, higher nymph mortality, decreased predation ability, and lower adult emergence rates in the F_1_ generation. Therefore, in integrated pest management (IPM) practices, it is recommended to avoid the combined use of dinotefuran with *P. lewisi*, or to minimize direct contact between natural enemies and pesticides during predation, such as through seed coating treatments. Alternatively, the use of safer insecticides that do not harm *P. lewisi*, or more environmentally friendly formulations, should be considered [12,30].

This study investigated the acute toxicity and transgenerational effects of dinotefuran on the predatory enemy *P. lewisi*, evaluating its impact on the reproductive performance, longevity of the parental generation, and the development and predatory ability of their offspring. The results showed that when both female and male adults are exposed to dinotefuran, the effects on *P. lewisi* and its offspring were most pronounced. Specifically, parental reproductive parameters such as the oviposition period, frequency and fecundity decreased by 72.73% to 86.59% compared to the control group. The emergence rate of offspring adults and their predatory capacity decreased by 50%, and the predation efficiency was reduced by 52.76% to 66.81%. The LC_50_ for dinotefuran was higher in female adults than in males. However, female adults were more severely affected under the same LC_50_ conditions. These findings provided valuable insights into the acute toxicity and transgenerational effects of dinotefuran on *P. lewisi*, which could inform ecological risk assessments related to pesticide exposure. Understanding these effects is crucial for the development of more balanced pest control strategies in IPM. This study highlights the need for careful consideration of chemical pesticide use, especially in combination with biological control agents. To minimize adverse effects on natural enemies, it is recommended that dinotefuran not be used concurrently with *P. lewisi* or within a short interval of its application. This research contributes to the theoretical foundation for balancing chemical and biological pest control, promoting their effective integration in pest management systems.

## Figures and Tables

**Table 1:** Pre-oviposition duration of *P. lewisi* parental generation (F_0_) exposed to dinotefuran.

Treatment	Pre-Oviposition Duration (d)
Both treated	30.40 ± 0.65 a
Female treated	22.30 ± 0.47 b
Male treated	16.00 ± 0.58 c
CK	11.50 ± 0.65 d

Different letters indicated statistically differences (*p* < 0.05) according to Turkey’s HSD Test.

**Table 2:** Formation proportion and female–male sex ratio of *P. lewisi* progeny (F_1_) adults under dinotefuran.

Treatments	Emergence Proportion of Adults (%)	Female–Male Sex Ratio
Both treated	0.50 ± 0.10 c	1:2
Female treated	0.60 ± 0.17 bc	1:1.57
Male treated	0.83 ± 0.06 ab	1:1.78
CK	1.00 ± 0 a	1:1.5

Different letters indicated statistically differences (*p* < 0.05) according to Turkey’s HSD Test.

**Table 3:** Functional responses of *P. lewisi* progeny (F_1_) to 3rd instar larvae of *S. exigua* under dinotefuran.

Treatments	Instar	Types	Functional Response Equation	*R^2^*	Instantaneous Attack Rate (*a*)	Handling Time (*T_h_*)/d	Daily Maximum Predition Number (1/*T_h_*)/Individual	Control Efficiency (*a*/*T_h_*)
Both treated	5th instar nymph	Type II	*N_a_* = 0.499*N*/(1 + 0.014*N*)	0.764	0.499 ± 0.016 b	0.029 ± 0.004 a	34.483 ± 4.864 a	17.207 ± 1.863 b
	Female adult	Type II	*N_a_* = 0.613*N*/(1 + 0.015*N*)	0.838	0.613 ± 0.022 a	0.024 ± 0.003 a	41.667 ± 5.305 a	25.542 ± 2.318 a
	Male adult	Type II	*N_a_* = 0.545*N*/(1 + 0.011*N*)	0.868	0.545 ± 0.026 b	0.021 ± 0.003 a	47.619 ± 6.968 a	25.952 ± 2.529 a
Female treated	5th instar nymph	Type II	*N_a_* = 0.560*N*/(1 + 0.015*N*)	0.839	0.560 ± 0.035 b	0.026 ± 0.005 a	38.462 ± 7.728 a	21.538 ± 2.921 b
	Female adult	Type II	*N_a_* = 0.649*N*/(1 + 0.014*N*)	0.911	0.649 ± 0.021 a	0.021 ± 0.002 a	47.619 ± 4.584 a	30.901 ± 1.964 a
	Male adult	Type II	*N_a_* = 0.583*N*/(1 + 0.011*N*)	0.900	0.583 ± 0.032 b	0.019 ± 0.002 a	52.632 ± 5.613 a	30.684 ± 1.566 a
Male treated	5th instar nymph	Type II	*N_a_* = 0.616*N*/(1 + 0.012*N*)	0.845	0.616 ± 0.051 b	0.020 ± 0.003 a	50.000 ± 7.701 a	30.800 ± 2.126 a
	Female adult	Type II	*N_a_* = 0.834*N*/(1 + 0.020*N*)	0.874	0.834 ± 0.035 a	0.024 ± 0.004 a	41.667 ± 7.176 a	34.750 ± 4.478 a
	Male adult	Type II	*N_a_* = 0.718*N*/(1 + 0.016*N*)	0.901	0.718 ± 0.036 b	0.022 ± 0.002 a	45.454 ± 4.172 a	32.636 ± 1.344 a
CK	5th instar nymph	Type II	*N_a_* = 0.985*N*/(1 + 0.019*N*)	0.882	0.985 ± 0.064 a	0.019 ± 0.004 a	52.632 ± 11.680 a	51.842 ± 7.954 b
	Female adult	Type II	*N_a_* = 1.024*N*/(1 + 0.015*N*)	0.918	1.024 ± 0.072 a	0.015 ± 0.002 a	66.667 ± 9.077 a	68.267 ± 4.393 a
	Male adult	Type II	*N_a_* = 0.925*N*/(1 + 0.013*N*)	0.911	0.925 ± 0.068 a	0.014 ± 0.001 a	71.429 ± 5.133 a	66.071 ± 0.139 ab

Different letters indicated statistically differences (*p* < 0.05) according to Turkey’s HSD Test.

**Table 4:** Functional responses of *P. lewisi* progeny (F_1_) to 3rd instar larvae of *S. litura* under dinotefuran.

Treatments	Instar	Types	Functional Response Equation	*R* ^2^	Instantaneous Attack Rate (*a*)	Handling Time (*T_h_*)/d	Daily Maximum Predition Number (1/*T_h_*)/individual	Control Efficiency (*a*/*T_h_*)
Both treated	5th instar nymph	Type II	*N_a_* = 0.459*N*/(1 + 0.013*N*)	0.834	0.459 ± 0.016 b	0.028 ± 0.004 a	35.714 ± 5.226 b	16.393 ± 1.813 b
	Female adult	Type II	*N_a_* = 0.519*N*/(1 + 0.008*N*)	0.902	0.519 ± 0.022 a	0.016 ± 0.003 b	62.500 ± 12.217 a	32.438 ± 4.907 a
	Male adult	Type II	*N_a_* = 0.498*N*/(1 + 0.010*N*)	0.855	0.498 ± 0.026 ab	0.020 ± 0.003 ab	50.000 ± 7.701 ab	24.900 ± 2.500 a
Female treated	5th instar nymph	Type II	*N_a_* = 0.539*N*/(1 + 0.013*N*)	0.857	0.539 ± 0.035 a	0.025 ± 0.005 a	40.000 ± 8.389 a	21.560 ± 3.053 b
	Female adult	Type II	*N_a_* = 0.611*N*/(1 + 0.011*N*)	0.922	0.611 ± 0.021 a	0.018 ± 0.002 a	55.556 ± 6.263 a	33.944 ± 2.643 a
	Male adult	Type II	*N_a_* = 0.593*N*/(1 + 0.014*N*)	0.884	0.593 ± 0.032 a	0.024 ± 0.002 a	41.667 ± 3.501 a	24.708 ± 0.732 b
Male treated	5th instar nymph	Type II	*N_a_* = 0.638*N*/(1 + 0.015*N*)	0.862	0.638 ± 0.051 b	0.024 ± 0.003 a	41.667 ± 5.305 a	26.583 ± 1.220 b
	Female adult	Type II	*N_a_* = 0.768*N*/(1 + 0.018*N*)	0.902	0.768 ± 0.035 a	0.023 ± 0.004 a	43.478 ± 7.836 a	33.391 ± 4.441 a
	Male adult	Type II	*N_a_* = 0.706*N*/(1 + 0.017*N*)	0.887	0.706 ± 0.036 ab	0.024 ± 0.002 a	41.667 ± 3.501 a	29.417 ± 0.959 ab
CK	5th instar nymph	Type II	*N_a_* = 0.958*N*/(1 + 0.019*N*)	0.909	0.958 ± 0.064 a	0.020 ± 0.004 a	50.000 ± 10.486 a	47.900 ± 6.690 b
	Female adult	Type II	*N_a_* = 1.030*N*/(1 + 0.015*N*)	0.901	1.030 ± 0.072 a	0.015 ± 0.002 a	66.667 ± 9.077 a	68.667 ± 4.447 a
	Male adult	Type II	*N_a_* = 0.956*N*/(1 + 0.014*N*)	0.895	0.956 ± 0.068 a	0.015 ± 0.001 a	66.667 ± 4.468 a	63.733 ± 0.286 a

Different letters indicated statistically differences (*p* < 0.05) according to Turkey’s HSD Test.

## Data Availability

The original contributions presented in this study are included in the article. Further inquiries can be directed to the corresponding author.
